# Supplementary material for: Virtual reality environment using a dome screen for procedural pain in young children during intravenous placement: A pilot randomized controlled trial
Source: PLoS One. 2021 Aug 31;16(8):e0256489. doi: 10.1371/journal.pone.0256489 (PMC8407539; doi:10.1371/journal.pone.0256489)
Supplement: S3 File — (DOCX) [file pone.0256489.s003.docx]

**Projector-based virtual reality dome environment for pain management in a pediatric emergency department**

**Version No: 1.1**

**Principal investigator**

**Name: Jung Wan Park**

**Department: Seoul national university hospital**

**Emergency medicine**

**Study Summary**

| Title | **Projector-based virtual reality dome environment for pain management in a pediatric emergency department** |
| --- | --- |
| Principal investigator | Jung Wan park |
| Sponsor organization | Seoul National University Hospital |

| Purpose | **We aimed to assess the feasibility and potential efficacy of a virtual reality environment using a dome screen for analgesia in young children during intravenous placement in the pediatric emergency department.** |
| --- | --- |
| Study design | Prospective randomized controlled study |
| Study periods | IRB approval date ~ 12 months |
| Population | Children aged 2 to 6 years undergoing intravenous placement at the pediatric emergency department of the Seoul National University Hospital. |
| Target number of participant | 20 |
| Vulnerable participant | Minors |
| Methods | After obtaining the written informed consent, children were randomized to either the intervention group or the control group according to the dates of their ED visits. Children whose ED visit date was an odd number were assigned to the intervention group, while those whose ED visit date was an even number were assigned to the control group. Children assigned to the intervention group experience the virtual reality environment using dome screen with a animation during the intravenous placement.  The child’s pain intensity was measured using the Face, Legs, Activity, Cry, and Consolability (FLACC) scale at each of the four time points of IV placement: Immediately after arrival at the blood collection room (base), immediately after the child lies in the bed (preparation), when the tourniquet is applied (tourniquet), and the moment at which the needle penetrates the skin (venipuncture). The guardian’s satisfaction and rating of the child’s distress were assessed using a 5-point Likert-type questionnaire. Feasibility and acceptability were assessed by asking pediatric emergency department staffs and emergency medical technicians who participated in the needle procedure about their satisfaction with the process of providing VR intervention to children as a distraction during the needle procedure. |
| Efficacy | Pain intensity measurement   - Using the Face, Legs, Activity, Cry and consolability (FLACC) scale   The guardian’s satisfaction and rating of the child’s distress   - Using a 5-point Likert-type questionnaire |
| Security | Complication related to intravenous placement such as pain, bleeding, infection may occur, but the risk does not differ depending on whether or not to participate in this study. In addition, children with hemodynamically unstable conditions or loss of consciousness are excluded from participation in this study. |
| Potential benefits | We expect VR using a dome screen to reduce distress (pain and anxiety) in children during intravenous placemen. |

**Proposal**

1. **Title**

Virtual Reality Environment Using a Dome Screen for Procedural Pain in Young Children during Intravenous Placement: A Pilot Randomized Controlled Trial

1. **Institution and adress**
   - Seoul National University Hospital
   - 101, Daehak-ro Jongno-gu, Seoul, Rep.of Korea
2. **Investigator**
3. **Principal investigator**
   - Joong Wan park, Professor
4. **Investigator**
   - Young Ho Kwak, Professor
   - Jae Yun Jung, Professor
   - Hyun Jung Jung, Fellow
   - So yun Kim, Fellow
   - Woo Ri bae, Fellow
5. **Researcher**
   - Hyo Jung Jin, Researcher
6. **Sponsor Organization**
   - Seoul national university hospital
7. **Expecting period of study**
   - IRB approval date t ~ 12 months
8. **Populations**
   - Children aged 2 to 6 years undergoing intravenous placement at the pediatric emergency department of the Seoul National University Hospital.
9. **Purpose**

Assess the feasibility and potential efficacy of a virtual reality environment using a dome screen for analgesia in young children during intravenous placement in the pediatric emergency department.

1. **Information of investigational drugs**
   - Non applicable
2. **Subject Eligibility**
3. **Inclusion criteria**
   - Children aged 2 to 6 years undergoing intravenous placement at the pediatric emergency department of the Seoul National University Hospital.
4. **Exclusion criteria**
   - Children were excluded from the study if 1) they needed urgent IV placement (e.g., due to an unstable hemodynamic condition or an altered mental status), 2) had developmental disabilities or facial anomalies that made it difficult to apply the pain scale, and if 3) their guardians had insufficient Korean language ability to understand the study protocol. 4) Children for whom IV catheter insertion failed on the first attempt were also excluded from the study.
5. **Sample size**
   - Similar to previous pilot studies regarding distraction methods for children during painful pro-cedures, formal sample size calculations were not required.
   - We aimed to recruit 20 children to provide sufficient preliminary evidence of the clinical efficacy of VR as a distraction method (intervention group [n=10] vs control group [n=10], respectively).
6. **Methods**
7. **Intervention**

- If the patient is eligible, PED staff explain the study and obtain written informed consent.
- Children are randomized to either the intervention group or the control group according to the dates of their ED visits.
- Five minutes before the emergency medical technician (EMT) start the IV placement, the child enter the blood collection room with his or her guardian, and the PED staff begin video recording. For the children assigned to the intervention group, a PED staff member then turn on the animation within 1 minute after the child enter the blood collection room. After the child lie down on a bed, the EMT began IV placement according to the following sequence: Tourniquet application, venipuncture site cleansing, venipuncture, indwelling IV cannula insertion. The guardian is allowed to hold the child’s opposite arm for reassurance during the procedure. Video recording is performed for approximately 2 minutes after venipuncture. After the child left the room, the guardian receive and complete a questionnaire about the needle procedure.
- Our VR equipment consisted of a dome screen developed by Dome & Dome Co. and a projector (EB-G7100, EPSON, Japan) linked to a personal computer playing the animated show ‘Pororo the Little Penguin’. The diameter and height of the dome screen are 1600 mm and 600 mm, respectively.
- We project the animation onto a dome screen using MadMapper version 4.0, a projection mapping software program, to provide the children with a VR environment.
- The animation used in this study is a famous Korean animated show that mainly targets children aged 3 to 5 years. We use a free downloadable episode available from the official ‘Pororo the Little Penguin’ channel on the online video sharing platform YouTube. Before the start of the study period, we received approval from the production company for the use of the episode for research purposes.
- Pain intensity assessments were completed by two PED staff members who were blinded to the study protocol and were based on video recordings. Two PED staff independently observed the video recording of the needle procedure for each child.
- The guardian’s satisfaction with the needle procedure were assessed using a 5-point Likert-type questionnaire after the needle procedure.

1. **Randomization**
   - If the patient was eligible, PED staff explained the study and obtained written informed consent. Children were randomized to either the intervention group or the control group according to the dates of their ED visits. Children whose ED visit date was an odd number were assigned to the intervention group with VR distraction, while those whose ED visit date was an even number were assigned to the control group.
2. **Outcomes**
   - Demographics

Child: Age, Sex, Reason for emergency department visit (Disease vs Trauma), Previous venipuncture (Yes vs No)

Guardian: Age, Relation, Number of children, Previous experience of observation of child’s procedure (Yes vs No)

- - Primary outcome

Pain intensity was measured using the Face, Legs, Activity, Cry, Consolability (FLACC) scale.

The FLACC scale is a validated pain measure for children who cannot report pain. The 5 elements of the FLACC scale are each scored on a range from 0-2 and then summed for a total score ranging from 0-10, with higher scores indicating greater pain intensity


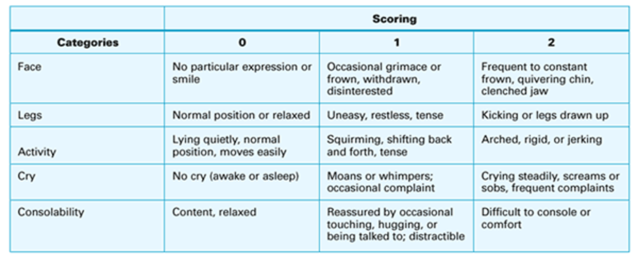


- - Secondary outcome

The guardian’s satisfaction with the needle procedure and rating of their child’s distress (pain, anxiety) were assessed using a 5-point Likert-type questionnaire after the needle procedure.

1. **Predictable side effect and benefit**
   - Common side effects of intravenous placement include pain, bleeding, infection.
   - Side effects associated with watching animated show via dome screen are not expected. Pain reduction can be expected by participating in this trial, but the exact benefit of participant has not been proven.
2. **Withdrawal**
   - Children who have failed to first try of intravenous placement, withdrawal to participate in the study, under sudden deterioration during the trial.
3. **The evaluation of safety including complication**

In case of sudden deterioration of children, trial should be stopped and appropriate treatment should be provided immediately. Adverse effect related to VR dome screen is reported to the IRB.

1. **Statistics**
   1. Categorical variables are reported as frequencies and percentages. Continuous variables are reported as medians and interquartile ranges (IQRs).
   2. The FLACC scale scores at each time point and the average of the FLACC scores at 3 time points (preparation, tourniquet, and venipuncture) were compared between the intervention group and control group using both the Mann-Whitney U test and the independent t-test.
   3. A between-groups comparison of the guardians’ responses to the postprocedural questionnaire was conducted using Fisher’s exact test.
   4. All statistical tests were performed at a significance level of 0.05 (2-sided) using STATA version 14.2 (StataCorp LP, College Station, TX, USA).
   5. The inter-rater reliabilities of scores of pain intensity by two PED staff are analyzed using interclass coefficients (ICCs).
2. **Ethics and regulation**
3. This study protocol conformed to the ethical guidelines of the 1975 Helsinki Declaration and International Conference on Harmonization of Technical Requirements of Pharmaceuticals for Human Use (ICH) Note for Guidance on Good Clinical Practice
4. There is no financial compensation for patients who participate in this study
5. For Vulnerable study subjects, minors aged 2 to 6 years old, a sufficient and easy explanation will be given, followed by oral consent. And written consent will be obtained from the guardian.
