# Supplementary material for: Virtual reality environment using a dome screen for procedural pain in young children during intravenous placement: A pilot randomized controlled trial
Source: PLoS One. 2021 Aug 31;16(8):e0256489. doi: 10.1371/journal.pone.0256489 (PMC8407539; doi:10.1371/journal.pone.0256489)
Supplement: S4 File — (DOCX) [file pone.0256489.s004.docx]

**소아응급실에서 정맥 혈관 확보 시 돔형 프로젝터 스크린 가상현실 환경을 이용한 통증 관리의 효과**

**Projector-based virtual reality dome environment for pain management in a pediatric emergency department**

**Version No: 1.1**

**책임연구자 소속: 서울대학교병원**

**책임연구자 이름: 박중완**

**연구 개요**

| 연구제목 | (국문) **소아응급실에서 정맥혈관 확보 시 돔형 프로젝터 스크린 가상현실 환경을 이용한 통증 관리의 효과** |
| --- | --- |
|  | (영문) **Projector-based virtual reality dome environment for pain management in a pediatric emergency department** |
| 책임연구자 | 응급의학과 박중완 교수 |
| 연구비 지원기관 | 서울대학교병원 |

| 연구 목적 | **2세에서 6세 사이의 어린 소아에서 돔형 프로젝터를 이용한 가상현실 환경을 이용하여 주의를 분산시키는 비약물적인 통증 조절 방법**을 시도함으로써, **정맥혈관 확보 시술 동안 환아의 통증과 불안이 감소되는 효과**가 있는지 알아보고자 함. |
| --- | --- |
| 연구 설계 | Prospective randomized controlled study |
| 연구 기간 | IRB승인일 ~ 12개월 |
| 연구 대상 | 응급실에 내원한 2~6세 환아 |
| 연구 대상자 수 | 20명 |
| 취약한 연구대상자 | 미성년자 |
| 연구 방법 | ○ 무작위로 환아를 대조군 혹은 시험군에 배정하며, 대조군의 경우 통상적인 과정에 따라 정맥혈관 확보 시술을 시행하고 시험군에 배정된 돔형 프로젝터를 이용한 가상현실 가상현실 환경 시청을 하며 정맥혈관 확보 시술을 시행하게 됨.  ○ 양 군 모두 시술 시행 과정에서 통증 점수 측정을 위하여 아이의 얼굴 및 전신의 모습을 동영상으로 촬영하고 촬영된 동영상은 연구의 과정과 목적을 모르는 제3의 연구자 2명이 각각 판독하여 **시술 전(base), 시술을 위한 자세를 잡을 때(position), 시술 시행 순간(puncture)** **환아의 통증(pain) (Primary outcome) 및 불안감 정도를 측정함**.  ○ 연구에 참여하는 모든 **환아의 시술 전(base), 시술을 위한 자세를 잡을 때(position), 시술 시행 순간(puncture) 심박수를 측정**하고 보호자 대상으로 설문하여 보호자가 느끼는 환아의 불안감 및 통증, **보호자 자신의 불안감과 만족도**를 측정함.  ○ 양 군의 **환아의 통증 및 불안감, 심박수 변화 및 보호자 설문 결과**를 통계적으로 분석하여 두 군 사이에 유의한 차이가 있는지 평가함. |
| 유효성 평가 | ○ 통증의 측정  - 잘 알려진 통증평가도구(FLACC)를 이용하여 시행  ○ 불안 정도 측정  - modified Yale preoperative anxiety scale  ○ 심박수 변화를 측정하여 통증 및 불안 정도의 간접적인 지표로 활용  ○ 보호자 만족도  - 5-point Likert scale 사용하여 연구 종료 전 설문 |
| 안전성 평가 | 통상적으로 정맥 혈관 확보 시에는 통증, 출혈, 감염 등의 합병증이 발생할 수 있으나 연구 참여 여부에 따라 위험도가 달라지지는 않음.  또한 쇼크 상태이거나 의식 저하 등의 긴급한 상황은 이 연구의 선정 대상에서 제외함. |
| 기대효과 및  예상결과 | 정맥 확보 시술에서 돔 스크린을 활용한 비약물적 진정을 통해 환아의 시술 중 통증 감소 및 불안감 감소 효과를 기대할 수 있음.  효과가 확인된 경우 아동 친화적인 소아 진료 환경 구축과 함께 환자/보호자의 만족도 제고를 달성할 수 있음. |

**연구계획서**

1. **연구 제목**
   - 소아응급실에서 정맥혈관 확보 시 돔형 프로젝터 스크린 가상현실 환경을 이용한 통증 관리의 효과
2. **연구 실시기관 명칭 및 주소**
   - 서울대학교병원, 서울특별시 종로구 대학로 101
3. **연구책임자 및 공동연구자 성명 및 직명**
4. **연구책임자:**
   - 박중완, 서울대학교병원 응급의학과 진료조교수
5. **공동연구자:**
   - 곽영호, 서울대학교병원 응급의학과 교수
   - 정재윤, 서울대학교병원 응급의학과 임상부교수
   - 정현정, 서울대학교병원 응급의학과 임상강사
   - 김소연, 서울대학교병원 응급의학과 임상강사
   - 배우리, 서울대학교병원 응급의학과 임상강사
6. **연구담당자**
   - 진효정, 서울대학교병원 응급의학과 연구원
7. **임상시험용 의약품 관리자**
   - 해당 없음
8. **임상시험용 의료기기 관리자**
   - - 해당 없음
9. **연구 의뢰기관
   1) 연구 의뢰기관 명칭 및 주소:** 해당 없음
   **2) 모니터요원 성명 및 직명:** 해당 없음
10. **연구비 지원기관 명칭 및 주소**
    - 서울대학교병원, 서울특별시 종로구 대학로 101
11. **예상연구기간**
    - IRB 승인 후 1년
12. **연구 대상 질환**
    - 응급실에 내원한 2~6세 사이의 소아 중 정맥 혈관 확보를 필요로 하는 환아
13. **연구의 배경 및 목적**

**1) 연구 배경**

○ 응급실을 방문하는 일은 어린이들과 보호자 모두에게 심한 스트레스이며, 많은 아이들이 통증을 이유로 방문하거나 응급실 체류 기간 중 통증을 유발하는 시술을 겪게 됨(Eldrige and Kennedy, 2010).

○ 정맥 혈관 확보는 응급실에서 비교적 흔히 시행되는 술기이며 바늘의 존재와 시술 과정이 환아에게 불안과 통증을 유발하여 아이에게 정신적 충격을 주거나 이후의 치료 과정을 어렵게 만들고, 보호자와 의료진의 관계가 악화되는 원인이 됨(Hughes, 2012 ; Crowley et al., 2011).

○ 응급실에서 소아의 통증 조절을 위하여 약물적인 방법 및 비약물적인 방법이 다양하게 연구되어 왔으며 상황에 따른 적절한 통증조절이 양질의 환자 관리를 위하여 필수적인 상황으로(Bailey and Trottier, 2016), 비약물적인 방법 중 장난감, 비디오게임 등을 이용하여 환아의 주의를 돌리는 방법이 최근 활발히 연구되고 있음(Koller and Goldman, 2012).

○ 가상현실은 실제와 유사한 가상의 세계를 구축하고, 현실과 가상현실과의 상호작용을 통해 사용자가 그 안에 있는 것처럼 체험 할 수 있도록 구현하는 것으로(Aguinas et al, 2001), 한층 더 몰입감 있는 주의 분산의 방법으로 주목받고 있음. 최근에는 소아 화상 환자에서 화상 드레싱 시 비약물적인 통증 조절 방법에 이용되어 효과가 있다는 연구 결과가 있음(Hoffman et al, 2014 ; Kipping et al, 2014).

○ 그러나 이전의 소아에서의 비약물적인 통증 조절은 주로 응급실이 아닌 환경에서 신생아나 미숙아에서 많이 이루어졌으며(de Sousa et al., 2008 ; Gray et al., 2000 ; Curtis et al., 2007), 응급실에서 연구가 이루어진 경우는 학동기 근처 연령의 아이들을 대상으로 한 연구가 많았음(Hartling et al, 2013 ; Wolyniez et al, 2013 ; Sinha et al, 2006 ; Miller et al, 2016).

○ 국내 소아 응급실의 주된 이용 연령층은 2~6세 사이의 아동들로(Choi et al, 2016) 해당 연령층에 대하여 이제까지 충분한 연구가 이루어지지 않은 상황임. 따라서 우리나라 소아응급실 진료의 질과 환아 및 보호자의 만족도를 높이기 위해서는 해당 연령층에 대하여, 응급실 내에서 가장 흔히 이루어지는 시술 중 하나인 정맥 혈관 확보의 통증 조절에 대한 대책 마련이 필수적임.

○ 또한 환아의 통증에 따라 보호자가 느끼는 불안감이 상승하기 때문에 (Smith et al, 2007) 환아의 통증 조절을 통하여 보호자의 심리 상태를 안정적으로 유지, 의료진과의 좋은 관계 확보 및 이후 치료 과정에서의 협조를 원활하게 할 수 있을 것임.

○ 기존의 가상현실을 통증 조절에 이용한 연구들의 대부분에서 가상현실 구현을 위해 HMD(Head Mounted Display) 장비가 필요하였고 (Atzori B et al., 2018 ; Atzori B et al., 2018 ; Piskorz J et al., 2018), 따라서 HMD 장비를 착용할 수 없는 어린 소아에서는 가상현실 시스템을 적용하기가 쉽지 않음.

○ 본 연구에서는 HMD 장비 없이 돔스크린을 이용하여 가상현실 시스템을 구현하여 이를 어린 소아의 정맥혈관 확보 과정에 적용하여 아이의 불안과 통증을 조절에 돔스크린 가상현실 환경의 효과를 알아보고자 함.

**2) 연구 가설 및 목적**
○ 연구 가설: 소아응급실에 방문하는 2~6세 환자가 정맥 혈관 확보를 받게 되는 경우 비약물적인 통증 조절 방법 중 돔형 스크린을 이용한 가상현실 환경을 통하여 주의를 돌리는 방법으로 통증 조절을 시도했을 때 기존의 방법과 비교하여 환아의 시술 중 통증 감소 및 불안감 감소, 보호자의 만족도 상승을 보일 것임.

1. **임상시험용 의약품의 정보 및 관리**
   - 해당 없음
2. **연구대상자의 선정 기준, 제외 기준, 목표한 대상자 수 및 산출 근거**
3. **선정기준**
   - 서울대학교병원 소아응급센터를 방문하는 2~6세 사이의 환자 중 정맥 혈관 확보를 받는 환자
4. **제외기준**
   - 발달 지연이나 안면 기형으로 환아의 통증 척도 파악이 어려운 경우
   - 쇼크 상태, 의식상태 저하 등으로 응급 정맥 혈관 확보가 필요한 경우
   - 보호자 동의를 받을 수 없는 경우 (한국어 의사소통이 불가한 경우 등)
   - 연구 수행 중 첫 정맥 혈관 확보 시도에서 실패한 경우
5. **목표한 대상자 수 및 산출 근거**
   - **본 연구는 Pilot study 로 진행할 예정으로 시험군(돔스크린 영상을 보면서 정맥혈관 확보) 10명, 대조군(돔스크린 영상 없이 정맥혈관 확보) 10명으로 총 20명의 환자를 대상으로 함.**
6. **연구 대상자 모집 계획**
   - 평일 10A~5P 중에서 연구원이 가능한 시간 동안 소아응급센터 내에 있는 2~6세의 환자들 중 정맥 혈관 확보가 필요한 환자가 있는 경우 제외 기준에 해당하는지 확인 후, 환자 및 보호자에게 연구에 대한 설명 후 동의를 구함.
   - 본 연구의 책임연구자는 인종이나 사회경제적 상태에만 근거해서 이 연구에 참여할 가능성이 있는 환자를 배제시키지 않을 것임. 이 연구의 선정기준에 합당하다면, 가능한 환자들이 이 연구에 참여할 수 있도록 모든 노력을 다할 것이며 본 기관 에서 치료받는 환자의 전체를 대표할 수 있도록 환자 보호자 들에게 연구의 목적을 주지시킬 것임.
   - 환자의 치료 의료진은 연구에 참여하지 않는 응급의학과 전공의로, 환자가 연구에 참여하지 않는다고 하여 치료에 불이익이 가해지지 않을 것이며, 기존의 치료를 진행할 것임. 연구 참여의 보상으로 금전 등을 주지 않음.
7. **연구 방법**
8. **구체적인 연구방법**

- 홀수날에 내원하여 연구에 동의한 환자는 시험군, 짝수날에 내원하여 연구에 동의한 환자는 대조군으로 배정
- 대조군에 배정된 환아는 응급실에서 통상적으로 하는 방법(환아는 침대에 눕고 침대 한쪽에서는 보호자가 환아를 잡고 있고 다른 한쪽에서는 응급구조사가 정맥혈관 확보)으로 정맥 혈관 확보를 받게 되며, 시험군에 배정된 경우 돔형 스크린을 통한 가상현실 동영상 시청을 하며 대조군에서와 마찬가지로 침대에 누워서 정맥 혈관 확보를 받게 됨.
- 돔형 스크린은 지름 1600mm, 높이 600mm 의 반구 형태로 제작
- 단초점 빔프로젝터를 이용하여 돔형 스크린에 영상을 프로젝션
- 동영상을 돔형 스크린에 입체적으로 프로젝션 하기 위해 비디오 맵핑 소프트웨어인 MadMapper (version 3.6.2)를 사용하여 동영상을 돔형 스크린에 왜곡을 최소화하여 입체 화면으로 구현함.
- 돔형 스크린을 통해 출력되는 동영상은 “핑크퐁 동요”로 저작권의 문제가 없도록 연구 시행 전 “핑크퐁” 업체의 지원 허가를 미리 받을 것임.
- 정맥 혈관 확보 시술 전 5분~시술 후 2분 간 연구에 참여하는 모든 환아의 전신을 비디오 촬영함.
- 대조군에 배정된 환아의 경우 기존의 정맥혈관 확보시와 마찬가지로 별도의 통증 조절 없이 비디오 촬영을 하며, 시험군에 배정된 환아의 경우 비디오 촬영이 시작된 후 1분 이내에 동영상 시청을 시작, 비디오 촬영 중단 1분 전 동영상 시청을 종료
- 이후 연구의 과정과 목적을 모르는 제3의 연구자 2인이 비디오를 판독하여 시술 전후 환아의 통증 변화를 측정
- 비디오 판독 시 눈가림 효과를 위해 음향이 없는 상태로 판독하게 함.
- 비디오 판독을 시행하는 2인은 연구의 목적과 내용을 전혀 알지 못하는 상태에서 판독을 시행하여 판독의 bias가 생기지 않게 함.
- 대조군, 시험군 환아 모두 시술 방에 들어온 직후(baseline, 방안에 환아와 보호자만 있는 상태), 시술을 위해 자세를 잡는 순간(아이를 침대에 눕히고 정맥을 찾는 과정, position), 정맥주사침을 찌르는 순간(puncture)에 각각의 순간의 통증 및 불안 점수를 통증평가도구(FLACC) 및 불안평가도구(mYPAS)를 사용하여 제3의 연구자 2인이 비디오 판독을 통해 측정.
- 2인의 연구자가 비디오판독을 통해 부여한 통증점수(FLACC) 및 불안점수(mYPAS)는 평균값을 통해 양군에서 비교할 예정임.
- 대조군, 시험군 환아의 심박수를 방에 들어온 직후(baseline, 방안에 환아와 보호자만 있는 상태), 시술을 위해 자세를 잡는 순간(아이를 침대에 눕히고 정맥을 찾는 과정, position), 정맥주사침을 찌르는 순간(puncture)에 측정
- 연구에 참여하는 모든 환아의 보호자는 자신의 시줄 중 불안감 및 시술 후 만족도, 주관적인 환아의 통증 및 불안감 정도를 5-point Likert scale로 보고함

1. **비교군 설정 및 무작위 배정 방법**

연구대상자의 선정 기준 에 부합하고, 제외 기준에 해당하지 않는 환자로서 무작위 법으로 각 군을 설정함. 홀수날 응급실을 내원한 환자는 시험군, 짝수날 응급실을 내원하여 연구에 참여한 환자는 대조군으로 정함.

1. **시험 의약품 투약 계획**
   - 해당 없음
2. **관찰항목, 임상검사항목 및 관찰검사방법**
   - 인구학적 변수
     1. 환아: 성별, 나이, 응급실 내원 이유(외상성, 비외상성), 이전 정맥 혈관 확보 시술 경험 유무
     2. 보호자: 성별, 나이, 환자와의 관계, 총 자녀 수, 이전 정맥 혈관 확보 시술 참관 경험 유무
   - 통증의 측정 (Primary outcome)
     1. 연구자에 의한 환아의 통증 측정은 FLACC (Face, Legs, Activity, Cry, Consolability)을 이용하여 시행함(그림2 참조). 해당 도구는 5가지 항목, 각 항목 당 2점 만점으로 총 10점 만점이며 이전 연구를 통해 0~7세 사이 소아의 시술시 통증 평가에 유용함이 입증됨(Nilsson et al, 2008 ; Babl et al, 2012).


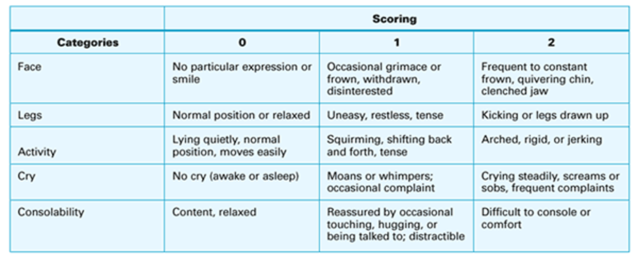


그림 2. FLACC 도구의 점수 배분

- - 환아의 불안감은 한국어 버전의 modified Yale Preoperative Anxiety Scale로 측정함. 상기 지표는 이전의 연구를 통해 한국어 버전의 신뢰성과 타당성이 검증이 되어 있으며(Jung et al, 2016), 응급실 내에서도 사용 가능함(Neville et al, 2016).
  - 환아의 심박수 변화를 함께 측정, 생리적 지표로 삼음.’
  - 보호자의 만족도 측정 · 5-point Likert scale을 사용하여 시술 과정의 만족도 보고

1. **기존 치료 및 연구와의 차별점**
   - 기존 정맥 혈관 확보 시술에서는 별도의 통증 조절 절차가 없이 시술을 진행하였음. 이 연구에서는 비교적 비용과 위험이 적은 비약물적인 방법으로 통증 조절을 시도하여 효과를 비교하려는 것임.
2. **연구대상자의 이익과 위험**
   - 일반적으로 정맥 혈관 확보 시술 시 가능한 부작용으로는 통증, 출혈, 감염 등이 있으며, 연구에 참여하는 경우 통증의 경감을 기대할 수 있겠으나 실제 연구 결과의 이익은 아직 증명되지 않았음. 가상현실 동영상의 시청으로 인한 위험은 특별히 예상되지 않음.
3. **중지∙탈락 기준**
   - 시험 과정 중 첫 정맥 혈관 확보 시도에서 실패한 경우
   - 시험 과정 중 혈압 저하, 의식 변화 등 급격한 상태의 악화로 눈가림 해제가 필요한 경우
   - 연구 대상자가 동의를 철회하는 경우
4. **부작용을 포함한 안전성의 평가기준, 평가 방법 및 보고 방법**
   - 각 군의 시험 과정 중 혈압 저하, 의식 변화 등 환아의 급격한 상태 악화가 있을 경우에는 즉시 실험을 중단하고 조치를 취할 것임. 다만 환아의 임상 경과 악화가 있더라도 동영상의 시청으로 인한 부작용은 아니며 환아의 병의 특성으로 생각되기 때문에 유해 사례 보고는 필요하지 않음.
5. **자료 분석 및 통계 계획**
   - 본 연구는 ITT 원칙으로 분석할 것임.
   - 나이, 체중(kg) 등의 수치형 자료는 평균, 표준편차, 혹은 중간값과 사분위수범위 들을 기술하고 두 군의 비교는 t-test 혹은 Mann-Whitney U test를 사용할 것임.
   - 범주형 자료는 그 비율을 제시하고 χ²-test 혹은 Fisher exact test를 사용
   - 각 검정에 대한 통계적 유의 수준은 5%로 설정
   - 시술 중-시술 전 통증 점수의 차이(FLACC) 및 불안감 점수의 차이(mYPAS)는 RMANOVA로 양 군을 비교하여 유의미한 차이 여부를 평가
   - 보호자의 만족도(5-point Likert scale)는 Mann-Whitney U test로 양 군을 비교하여 유의한 차이 여부를 평가
   - 비디오 판독시 연구자간의 일치도는 ICC(Intraclass Correlation Coefficient)를 구하여 평가
6. **자료 관리 계획**
   - 연구 수행 연구원이 자료 기록 및 수집 후 암호화된 엑셀 파일로 보관하여 연구자 외 자료의 접근이 불가능하게 함.
   - 연구 자료의 관리는 책임연구자가 최종 관리함.
7. **임상시험 후 연구대상자의 진료 및 치료기준**
   - 연구대상자의 임상시험 후 진료 및 치료는 기존의 계획대로 진행함.
   - 임상시험이 연구대상자의 향후 진료 및 치료에 영향을 끼치지 않음
8. **연구수행일정표**

| 연 구 내 용 | 추 진 일 정(기간) | | | | | | | | | | | | 비 고 |
| --- | --- | --- | --- | --- | --- | --- | --- | --- | --- | --- | --- | --- | --- |
|  | 1 | 2 | 3 | 4 | 5 | 6 | 7 | 8 | 9 | 10 | 11 | 12 |  |
| IRB 획득 | ○ | ○ |  |  |  |  |  |  |  |  |  |  |  |
| 연구 프로토콜 공유 |  | ○ |  |  |  |  |  |  |  |  |  |  |  |
| 환자군 모집 및 비디오 촬영 |  |  | ○ | ○ | ○ | ○ | ○ | ○ |  |  |  |  |  |
| 비디오 판독 |  |  | ○ | ○ | ○ | ○ | ○ | ○ | ○ |  |  |  |  |
| 통계 및 정리 |  |  |  |  |  |  |  |  | ○ | ○ | ○ | ○ |  |
| 추 진 진 도 (%) | 5 | 10 | 15 | 30 | 40 | 50 | 60 | 70 | 80 | 90 | 95 | 100 |  |

1. **자료 및 안전성 모니터링 계획 (Data and Safety Monitoring Plan)**
   - 최소위험연구로 해당 없음
2. **연구대상자의 안전보호를 위한 대책**
3. **연구의 윤리성 확보를 위한 기본 방안**
   - 본 연구는 헬싱키 선언 및 ICH-GCP를 준수하여 진행될 것이며, IRB 승인 후에 연구가 시작될 것임. 본 연구에 포함되는 연구대상자들의 기초 자료들은 익명으로 다루어지며, 모두 엄격하게 비밀이 유지되어 항상 보호를 받게 됨.
4. **연구대상자의 동의 과정**
   - 연구대상자는 연구원이 확인.
   - 연구원은 연구책임자 및 공동연구자에게 연락함.
   - 연구책임자 또는 공동연구자가 환아 보호자에게 연구 전반의 진행 사항 및 예견되는 부작용 등에 대해 충분히 설명할 것이며 환아에게는 그림 등으로 구성된 연구 설명문을 통해 구두 승낙을 받을 것이며 보호자에게는 서면 동의서를 받음.
   - 환아 보호자에게 30분 내외의 동의서를 읽을 충분한 시간을 주고, 서명 전 문의사항에 응답할 수 있도록 함.
   - 연구 설명과 동의 취득 과정 사이의 대기 시간에는 일반적으로 필요한 검사들이 시행됨.
   - 설명 중 또는 동의 이후 연구 진행 중에 연구 대상자나 보호자가 연구 진행을 원치 않는 경우 어떤 경우라도 동의 철회를 할 수 있도록 함.
   - 연구에 참여하지 않는 경우에는 일상적으로 제공되는 치료가 환자를 담당한 의사에 의해 제공됨.
   - 동의를 받을 때에는 일반적인 대한민국의 성인이 사용하는 언어를 사용하여 동의를 받는다.
   - 연구동의서 사본을 보호자에게 제공한다.
5. **연구대상자의 보상 방안**
   - 사례와 보상은 없음
6. **연구대상자의 개인정보보호 방안**
   - 수집된 자료는 연구 대상 환자의 프라이버시를 보호하는 차원에서 허가된 연구자 이외의 사람에게 열람, 유출되지 않도록 함.
   - 연구 대상 환자에 대한 자료는 증례기록지로 수집하여 수집된 자료는 이중 시건 장치를 하여 보관함.
   - 연구 번호(Serial number)와 개인 정보에 대한 자료는 연구 대상 환자에 대한 자료와 분리하여 비밀번호가 설정된 상태로 입력하고 보관함으로써 환자의 개인정보가 유출되지 않도록 할 것이며 추후 외래 추적관찰 혹은 전화 조사가 필요한 경우에만 병록 번호 및 개인정보를 통해 확인 할 예정임.
   - 연구 관련 기록은 연구가 종료된 시점부터 3년간 보관할 것이며, 보관기관이 지난 문서 중 개인정도에 관한 사항은 개인정보보호법 시행령 제 16조에 따라 파기할 것임.
7. **취약한 연구대상자를 포함하는 경우 추가적인 보호조치 방안**
   - 본 연구는 2세~6세의 미성년자가 포함되는 연구로 환아에게는 충분하고 쉬운 설명 후 구두 동의를, 보호자에게는 서면 동의서를 받을 것임.
8. **인체유래물의 보관 및 폐기 방법**
   - 해당 없음
9. **참고 문헌**
   - Eldridge C, Kennedy R. Nonpharmacologic techniques for distress reduction during emergencyㅜ medical care: a review. Clin Pediatr Emerg Med 2010;11:244–50.
   - Hughes T. Providing information to children before and during venepuncture. Nurs Child Young People 2012;24:23–8.
   - Crowley M, Storer A, Heaton K, et al. Emergency nursing resource: needle-related procedural pain in pediatric patients in the emergency department. J Emerg Nurs. 2011;37:246–251.
   - Bailey B, Trottier E.D. Managing Pediatric Pain in the Emergency Department, Paediatr Drugs 2016;18;4;287-301.
   - Koller D, Goldman RD. Distraction techniques for children undergoing procedures: a critical review of pediatric research. J Pediatr Nurs 2012;27:652–81.
   - Aguinas H, Henle CA, Beaty JC., Jr Virtual reality technology: a new tool for personnel selection. Int J Sel Assess 2001;9:70–83.
   - Hoffman HG, et al., Feasibility of articulated arm mounted Oculus Rift Virtual Reality goggles for adjunctive pain control during occupational therapy in pediatric burn patients. Cyberpsychol Behav Soc Netw 2014;17:397-401.
   - Kipping B, Rodger S, Miller K, Kimble RM, Virtual reality for acute pain reduction in adolescents undergoing burn wound care: a prospective randomized controlled trial. Burns 2012;38:650-7
   - de Sousa Freire NB, Santos Garcia JB, Carvahlo Lamy Z. Evaluation of analgesic effect of skin-to-skin contact compared to oral glucose in preterm neonates. Pain 2008;139:28-33.
   - Gray L, Watt L, Blass EM. Skin-to-skin contact is analgesic in healthy newborns. Pediatrics. January 2000;105:e14.
   - Curtis SJ, Jou H, Ali S, Vandermeer B, Klassen T. A randomized controlled trial of sucrose and/or pacifier as analgesia for infants receiving venipuncture in a pediatric emergency department. BMC Pediatr 2007;7:27.
   - Hartling L, Newton A.S, Liagn Y, et al. Music to reduce pain and distress in the pediatric emergency department: a randomized clinical trial. JAMA Pediatr 2013;167;826-35.
   - Wolyniez I, Rimon A, Scolnik D, et al. The effect of a medical clown on pain during intravenous access in the pediatric emergency department: a randomized prospective pilot study. Clin Pediatr 2013;52;1168-72.
   - Sinha M, Christopher N. C, Fenn R, Reeves L. Evaluation of nonpharmacologic methods of pain and anxiety management for laceration repair in the pediatric emergency department Pediatrics 2006;117;1162-68.
   - Miller K, Tan X, Hobson AD, et al. A Prospective Randomized Controlled Trial of Nonpharmacological Pain Management During Intravenous Cannulation in a Pediatric Emergency Department. Pediatr Emerg Care 2016;32;444-51.
   - Choi S, Cho I, Ha C. Epidemiologic characteristics of children who visited an emergency department: a single center study over three years. PEMJ 2016;3;24-31.
   - Smith, RW, Shah V, Goldman, RD, Taddio A. Caregiver’s response to pain in their children in the emergency department. Arch Pediatr Adolesc Med 2007;161;578-82.
   - Atzori B, Hoffman HG, Vagnoli L, et al. Virtual Reality Analgesia During Venipuncture in Pediatric Patients With Onco-Hematological Diseases. Front Psychol 2018;9:2508.
   - Atzori B, Lauro Grotto R, Giugni A, et al. Virtual Reality Analgesia for Pediatric Dental Patients. Front Psychol 2018;9:2265.
   - Piskorz J, Czub M. Effectiveness of a virtual reality intervention to minimize pediatric stress and pain intensity during venipuncture. J Spec Pediatr Nurs 2018;23.
   - Nilsson S, Finnstrom B, Kokinsky E. The FLACC behavioral scale for procedural pain assessment in children aged 5-16 years. Paediatr Anaesth 2008;18;767-74.
   - Babl FE, Crellin D, Cheng J, Sullivan TP, O'Sullivan R, Hutchinson A. The use of the faces, legs, activity, cry and consolability scale to assess procedural pain and distress in young children. Pediatr Emerg Care 2012;28;1281-96.
   - Jung K, Im MH, Hwang JM, et al. Reliability and validity of Korean version of modified: Yale preoperative anxiety scale. Ann Surg Treat Res 2016;90;43-8.
   - Neville, DN, Hayes KR, Ivan Y, McDowell ER, Pitetti RD. Double-blind Randomized Controlled Trial of Intranasal Dexmedetomidine Versus Intranasal Midazolam as Anxiolysis Prior to Pediatric Laceration Repair in the Emergency Department. Acad Emerg Med 2016;23;910-7.
